# Autism spectrum disorder in tuberous sclerosis complex: searching for risk markers

**DOI:** 10.1186/s13023-015-0371-1

**Published:** 2015-12-02

**Authors:** Aglaia Vignoli, Francesca La Briola, Angela Peron, Katherine Turner, Chiara Vannicola, Monica Saccani, Elisabetta Magnaghi, Giulia Federica Scornavacca, Maria Paola Canevini

**Affiliations:** Child Neuropsychiatry Unit – Epilepsy Center, AO San Paolo, Department of Health Sciences, Università degli Studi di Milano, Milan, Italy; Child Neuropsychiatry Unit, AO San Paolo, Milan, Italy

**Keywords:** Tuberous Sclerosis Complex, TSC, Autism Spectrum Disorder, ASD, Autism, Social Communication Questionnaire, TSC1, TSC2

## Abstract

**Background:**

Neuropsychiatric disorders are present in up to 90 % of patients with Tuberous Sclerosis Complex (TSC), and represent an important issue for families. Autism Spectrum Disorder (ASD) is the most common neurobehavioral disease, affecting up to 61 % of patients. The aims of this study were: 1) to assess the prevalence of ASD in a TSC population; 2) to describe the severity of ASD; 3) to identify potential risk factors associated with the development of ASD in TSC patients.

**Methods:**

We selected 42 individuals over age 4 years with a definite diagnosis of TSC and followed at a TSC clinic in Northern Italy. We collected and reported clinical and genetic data, as well as cognitive level, for each of them. We administered the Social Communication Questionnaire (SCQ) as a reliable screening tool for ASD, and performed comparisons between the average scores and each clinical and genetic feature.

**Results:**

Seventeen out of 42 patients (40.5 %) had a score at the SCQ suggestive of ASD (≥15 points). When calculated for each cognitive level category, the average SCQ score tended to be progressively higher in patients with a worse cognitive level, and the number of pathological SCQ scores increased with worsening of intellectual disability. With respect to ASD severity, the scores were equally distributed, indicating that the degree of ASD in TSC patients may have a large variability. By comparing the average SCQ scores with the clinical features, we found statistically significant correlations with epilepsy, seizure onset before age one year, spasms, mutations in *TSC2*, cognitive level, sleep disorders, and other psychiatric problems, but not with seizure frequency, tubers localization and gender.

**Conclusions:**

Our study showed a prevalence of ASD of 40.5 %, confirming the higher risk for this disorder in patients with TSC. However, the severity seems to have a notable variability in TSC patients. Risk factors for ASD are epilepsy, infantile spams, and mutations in *TSC2*.

## Background

Tuberous Sclerosis Complex (TSC) is a rare disorder associated with multiorgan involvement, including the brain, kidneys, heart, lung, skin and eyes. Patients may also be affected by behavioral, intellectual, psychiatric and psychosocial difficulties, which often represent the greatest burden of this disease, for both families and physicians. The Neuropsychiatric Panel at the 2012 International Tuberous Sclerosis Complex Consensus Conference adopted the umbrella term TAND (TSC-associated neuropsychiatric disorders) to describe these issues [1]. Recently, a specific screening tool (the TAND Checklist) has been developed in order to raise awareness of these problems, which can affect 90 % of patients [2].

Among the psychiatric problems associated with TSC, autism spectrum disorder (ASD) is the most common, affecting up to 61 % of patients [3]. However, its severity is not frequently reported, and the risk factors for ASD in the TSC population are still controversial. Epilepsy is one of the major issues in TSC [4] and is considered a potential risk factor for ASD in TSC patients, especially when epilepsy starts early and with infantile spasms. Several reports have indicated infantile spasms as risk factors for developing autism in children with TSC [5, 6]. However, infantile spasms are not a sufficient cause of ADS, suggesting that a common neurobiological mechanism can lead to both infantile spasms and ASD [7]. The localization of cortical tubers was identified as another risk factor for developing ASD in TSC patients: both frontal and temporal tubers were associated with ASD, and cyst-like cortical tubers were more common in patients with TSC and ASD [8]. Finally, intellectual disability (ID) is considered another risk factor for ASD in patients with TSC [5, 9]. Interestingly, correlations between ASD and genotype are emerging, and *TSC2* mutations seem to be more frequently associated with ASD, regardless the mutation type [8].

Overall, knowledge about the characteristics of ASD as an endophenotype in TSC needs to be improved, and a reliable and valid assessment is needed in this population [3]. For these reasons, the aims of this study were: 1) to assess the prevalence of ASD in a TSC population; 2) to describe the severity of ASD; 3) to identify potential risk factors associated with the development of ASD in TSC patients.

## Methods

### Participants

We selected 42 individuals with a definite diagnosis of TSC [10] among 113 patients who underwent a follow-up visit in the TSC clinic at San Paolo University Hospital in Milan, Italy, between September 2013 and July 2014. According to the inclusion criteria, the patients had to be at least 4 years old and have a mental age over 2 years. Patients over 18 years were included only if they resulted to be dependent on a caregiver.

Seventy-one patients were excluded as they did not meet the inclusion criteria: ten were under age four, 18 showed profound ID with a mental age under 2 years, 41 were adults not dependent on a caregiver, for two individuals the caregiver was not present at the time of the visit. Nobody declined to participate in the study.

All the patients included in this study underwent a cognitive and communication skills/social functioning evaluation. We collected data on each patient’s clinical history, epilepsy, and genetic tests as well.

Informed consent was obtained, and this study was approved by the IRB of our institution.

### Cognitive evaluation

Cognitive level was assessed using the following tests, based on the patient’s age and the test’s applicability:

- Griffiths scale and Wechsler scale (*Wechsler preschool and primary scale of intelligence, revised*, WPPSI-R) for patients aged 3 to 6 years;
- Wechsler scale (*Wechsler intelligence scale for children, Third edition* WISC-III) and Raven’s colored progressive matrices for patients aged 6 to 12 years;
- Wechsler scale (WISC-III, *Wechsler Adult Intelligence Scale, revised,* WAIS-R), Leiter-R scale, and Raven’s colored progressive matrices for patients aged 13 to 18 years;
- Wechsler scale (WAIS-R) for adult patients when possible, and Raven’s standard progressive matrices in one case.

Cognitive level was evaluated clinically when a formal test could not be performed due to severely impaired mental status, according to the international statistical classification of diseases and related health problems, tenth revision (ICD-10) [11].

### Communication skills and social functioning evaluation

We used the Social Communication Questionnaire (SCQ), lifetime form, Italian translation [12], in order to evaluate communication skills and social functioning of each patient, as a quick and reliable screening tool for ASD [13]. The questionnaire is composed of 40 yes/no questions adapted from the *Autism Diagnostic Interview* (ADI-R). Most of the questions correspond to the three main ADI domains: 1. Reciprocal social interaction; 2. Language/communication; 3. Restricted, repetitive, and stereotyped behaviors and interests. According to the instructions, the SCQ was completed by the parents/caregivers. Although it could be filled in autonomously, we chose to let caregivers complete it in the presence of a physician or psychologist since clinical experience has shown this method leads to more accurate results. A clinical cut-off of 15 points or more is suggestive of ASD, with a sensitivity of 0.85, a specificity of 0.75, positive predictive value of 0.93, and negative predictive value of 0.55 when differentiating ASD from other diagnoses [14]. A score of 22 points or higher is suggestive of autism (AD), differentiating it from other forms of ASD [15]. A team of child neurologists with wide expertise in ASD evaluated clinically all the patients. The diagnosis of ASD/AD was confirmed or excluded clinically according to the DSM-IV-TR criteria. Quality of the social interactions (social overtures, social response, shared enjoyment in interaction, unusual eye contact, facial expressions, facial expressions, reciprocal smiling at others, giving, showing, spontaneous initiation of joint attention), communicative behaviors (pointing, gestures, use of other’s body to communicate) and stereotyped behavior and restricted interests (unusually repetitive and stereotyped behaviors, unusual sensory interest in play material/person, hand and finger mannerisms, other complex mannerisms or stereotyped body movements) were evaluated.

### Statistical analysis

Data on the 42 patients were transferred into an electronic database and processed using the Statistical Package for the Social Sciences (SPSS, IBM, Chicago, IL, U.S.A.) for Macintosh, version 21.0. Continuous variables were presented as mean and standard deviation (SD). We performed comparisons between the average SCQ scores for each clinical and genetic feature, using *χ*2 test (categorized variables not normally distributed), independent sample two tailed Student (t-tests, variables with a normal distribution) and one-way analyses of variance (ANOVA) with Bonferroni post hoc as appropriate.

We chose a two-tailed p value of 0.05 or less to define statistically significant results.

## Results

We analyzed 42 patients with a definite diagnosis of TSC. This cohort was composed of 24 females (57.1 %) and 18 males (42.9 %), aged 4 to 44 years, with a median age of 19.3 years. Twenty-three of them (54.8 %) were adults and 19 (45.2 %) were children under 18 years of age. With respect to genetic analyses, 10 patients (23.8 %) had a mutation in *TSC1*, 30 (71.4 %) a mutation in *TSC2*, and in two patients (4.8 %) no mutations in either gene could be identified by current diagnostic methods (NMI).

The prevalence of cortical tubers, subependimal nodules (SENs), subependimal giant cell astrocytomas (SEGAs), sleep disorders, psychiatric symptoms, and epilepsy was assessed, resulting consistent with the data present in the scientific literature (data not shown).

We were able to measure the cognitive level in 30 out of 42 patients. The remaining 12 individuals were considered to be non-testable due to a severely impaired mental status (10) or poor compliance (2): therefore, a child neurologist evaluated their cognitive level clinically. The results are shown in Table 1.

**Table 1:** Cognitive levels of the population presented in this study

Cognitive level	Number of patients (%)
Normal	5 (11.9 %)
BIF	6 (14.3 %)
Mild ID	9 (21.4 %)
Moderate ID	12 (28.6 %)
Severe ID	10 (23.8 %)

*BIF* Borderline Intellectual Functioning; *ID* Intellectual Disability

**Fig. 1:**
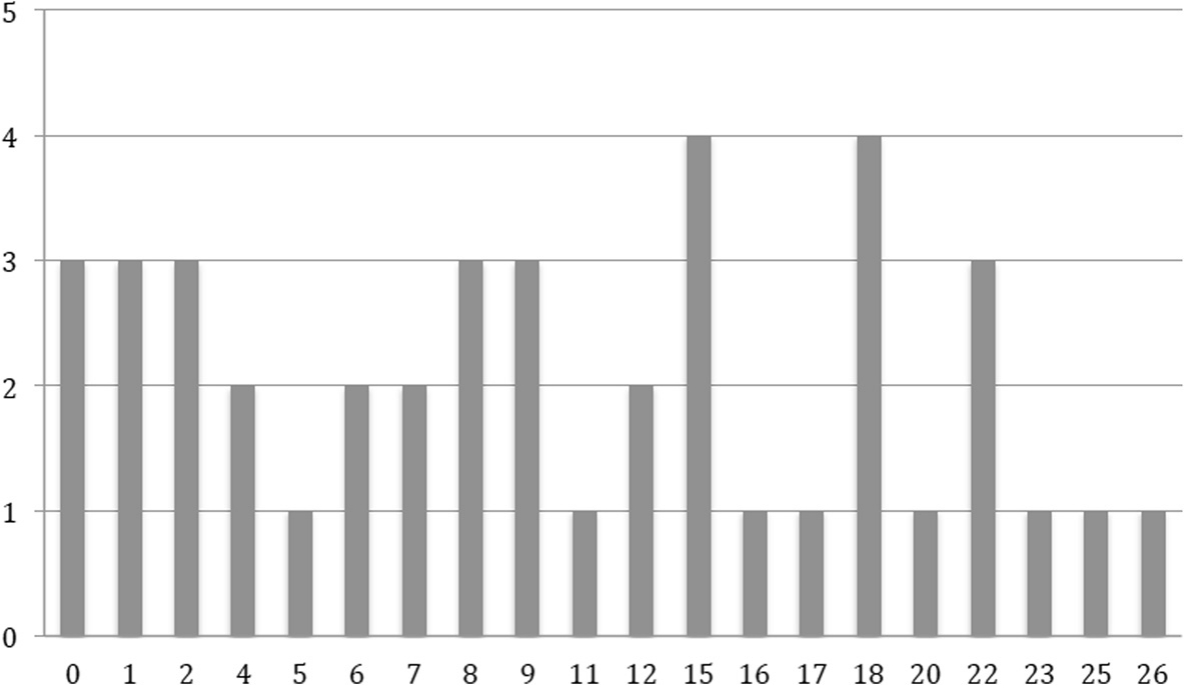
Distribution of SCQ scores. The horizontal axis displays the scores, and the vertical axis shows the number of patients

**Fig. 2:**
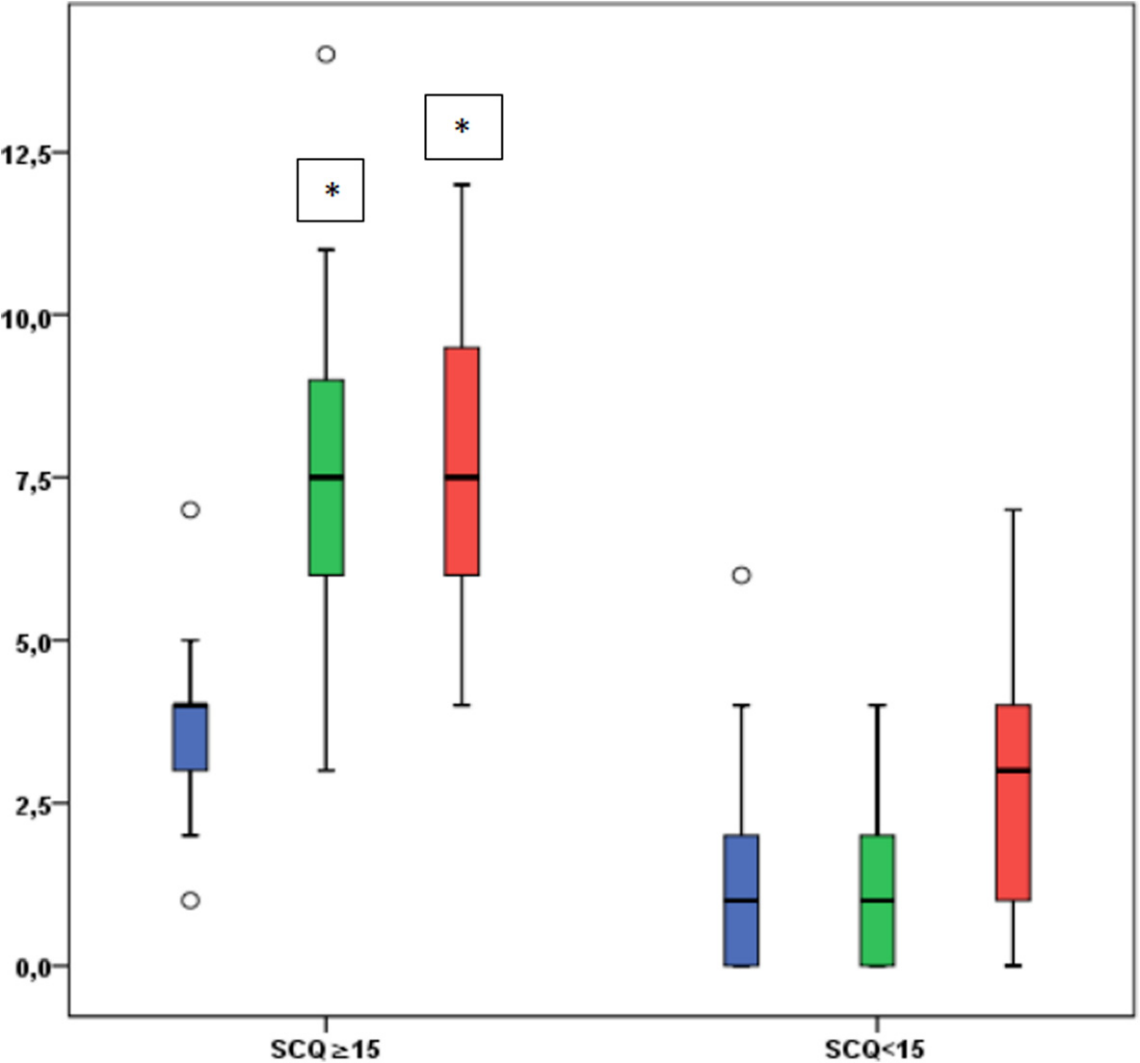
Ranges of the scores obtained in each of the three ADI domains in the patients with a score ≥ 15 (*left*) and in those with a score < 15 (*right*). Blue: restricted, repetitive, and stereotyped behaviors and interests. *Green*: reciprocal social interaction. *Red*: language/communication

**Fig. 3:**
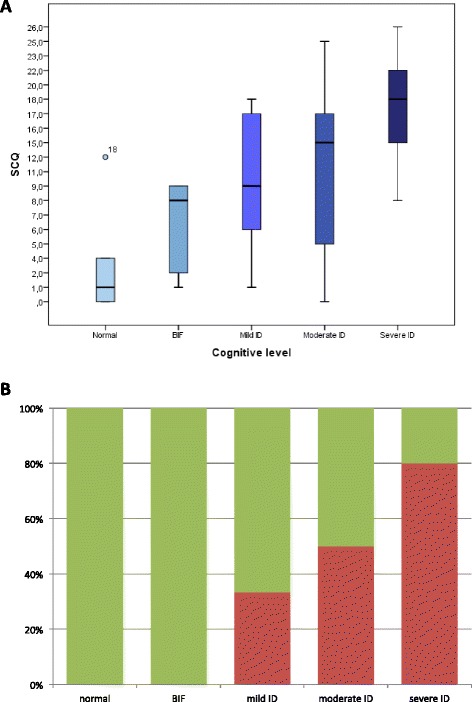
A:SCQ scores in relation to cognitive level. Note how the score progressively increases with worsening of mental status. B: Proportion between normal (<15) and pathological (≥15) SCQ scores according to cognitive level. *Green rectangles* indicate normal scores, and red rectangles indicate pathological scores. BIF: Borderline Intellectual Functioning. ID: Intellectual Disability

With respect to the SCQ, 17 patients (40.5 %) had a score of 15 points or higher, and 25 (59.5 %) had a score lower than 15. The scores were equally distributed in our sample, as shown in Fig. 1. A comparison between the score ranges obtained in each of the three ADI domains in the patients with a score ≥ 15 and in those with a score < 15 is shown in Fig. 2. The total average score was 10.9 (SD 7.8), ranging 0–26 points. When calculated for each cognitive level category, the average SCQ score tended to be progressively higher in patients with a worse cognitive level: 9.8 (±6.7) in patients with mild ID, 11.75 (±8.1) in patients with moderate ID, and 17.7 (±6.2) in patients with severe ID (Fig. 3a). All the patients with a normal or borderline mental status scored less than 15. The number of pathological SCQ scores (≥15) increased with worsening of intellectual disability as well (Fig. 3b). By comparing the scores obtained in the three main domains with the cognitive level, we found statically significant differences with respect to social interaction (*p* = 0.003) and communication (*p* = 0.007), but not to repetitive behaviors.

In order to better characterize the patients with an SCQ score suggestive of ASD, we evaluated at first the demographic, clinical and genetic characteristics in these patients:

### Demographic features

Six were children (35.3 %) and 11 were adults (64.7 %), aged 6–44 years (median age 21.5 years). The gender distribution was almost equal: 58.8 % females versus 41.2 % males. Only three patients (17.6 %) had a positive family history of TSC.

### Genetics

Fifteen patients (88.2 %) had a mutation in *TSC2*, two (11.8 %) had a mutation in *TSC1*. No NMI patient had a pathological score. Among the TSC2 patients, 12 had a protein truncating (PT) mutation, two a non-truncating (NT) mutation in the GAP domain, and one a proximal NT mutation but not in the Hamartin Interaction Domain.

### Central nervous system features

All of them but one presented with multiple cortical tubers; 15 (88.2 %) had SENs; four (23.5 %) developed a SEGA. With respect to tubers localization, there was not a prevalence of a lobe to the others.

### Epilepsy

Seizures were present in all the patients: mean age at onset was 7.9 months (birth to 18 months), with the majority of patients having a positive history for infantile spasms (11 patients, 64.7 %), and still having seizures at the time of the evaluation (11 patients, 64.7 %). Most of them were on multiple antiepileptic drugs (11 patients, 64.7 %). EEG was normal in three patients (17.6 %), while it showed focal activity in 12 (70.6 %), and a slow background activity in seven (41.2 %).

### Sleep disorders

Seven patients (41.2 %) presented with a sleep disorder.

### Psychiatric diseases

Ten patients (58.8 %) showed psychiatric symptoms (OCD, anxiety, psychosis, mood changes) or behavioral anomalies (hyperactivity, aggressive behavior).

Since the SCQ provides a quantitative measurement of symptom severity, we examined the total scores for each patient with a pathologic SCQ. The scores ranged from 15 to 26 points, with the most represented ones being 15 (four patients), 18 (four patients), and 22 (three patients), and the other scores being equally distributed. Six out of 17 patients (35 %) scored 22 points or higher. In all of them we confirmed a diagnosis of autism clinically, according to the DSM-IV-TR criteria. We also excluded the diagnosis of ASD in all the individuals with SCQ scores less than 15 after clinical evaluation by an expert.

Finally, we considered the clinical and genetics characteristics of all the 42 patients, calculated the average SCQ score for each of them, and compared the scores for each variable in order to look for possible risk factors for ASD. The results are shown in Tables 2 and 3.

**Table 2:** Comparison between the average SCQ scores for each clinical and genetic feature

Gender	Male		Female	
Number of patients	18 (42.8 %)		24 (57.1 %)	NS
Average SCQ score ±SD	10.6 ± 7.0		11.2 ± 8.5	
Mutation	TSC1	TSC2	NMI	
Number of patients	10 (23.8 %)	30 (71.4 %)	2 (4.8 %)	
Average SCQ score ±SD	6.3 ± 6.0	12.6 ± 8.0	9.5 ± 3.5	NS p<0.03*
	6.3 ± 6.0	12.6 ± 8.0		
Epilepsy	Present		Absent	
Number of patients	36 (85.7 %)		6 (14.3 %)	
Average SCQ score ±SD	12.1 ±7.7		4.2 ± 4.7	p<0.05*
Onset of seizures	Before age 12 months		After age 12 months	
Number of patients	25 (69.4 %)		11 (30.6 %)	
Average SCQ score ±SD	14.0 ± 7.4		7.5 ± 6.4	p<0.05*
Spasms	Present		Absent	
Number of patients	18 (50 %)		18 (50 %)	
Average SCQ score ±SD	15.1 ±7.4		8.2 ± 6.6	p<0.05*
AEDs (at time of evaluation)	Monotherapy		Polytherapy	
Number of patients	13 (39.4 %)		20 (60.6 %)	
Average SCQ score ±SD	10.1 ± 9.0		13.6 ± 6.3	NS
Seizure frequency	None	Monthly	Weekly	
Number of patients	19 (52.8 %)	7 (19.4 %)	10 (27.8 %)	
Average SCQ score ±SD	9.9 ± 8.0	15.7 ± 5.3	13.6 ± 7.8	NS
IQ	IQ≥70		IQ<70	
Number of patients	11 (36.7 %)		19 (63.3 %)	
Average SCQ score ±SD	4.8 ± 4.3		11.5 ±6.7	p<0.05*
Sleep disorders	Present		Absent	
Number of patients	9 (21.4 %)		33 (78.6 %)	
Average SCQ score ±SD	16.4 ± 8.0		9.4 ± 7.1	p<0.05*
Psychiatric disorders	Present		Absent	
Number of patients	17 (41.5 %)		24 (58.5 %)	
Average SCQ score ±SD	13.3 ± 8.7		8.6 ± 6.0	p<0.05*

Statistically significant findings are written in bold

*NS* not statistically significant; *AEDs* antiepileptic drugs

**Table 3:** This table shows the number of patients with *TSC1* and *TSC2* mutations for each group (pathologic and non-pathologic SCQ score)

	TSC and SCQ≥15	TSC and SCQ<15	*p* value
TSC1	2	8	0.05
TSC2	15	15	1.00
NMI	0	2	not performed
total	17	25	

Note that TSC1 patients have statistically significant less frequency of pathologic SCQ

## Discussion

TSC is a complex genetic disease with multiple difficulties. Neurobehavioral problems often represent the major issue in affected individuals and their families. Patients with TSC should undergo a comprehensive phenotypic evaluation throughout measures of neural activity (EEG), detection of subtle structural abnormalities (MRI), and appropriate behavioral assessment [3].

Besides the application of structured screening tools, such as the TAND checklist [2], deeper insight for specific neuropsychiatric disabilities should be implemented in the TSC population. Recently, Granader et al. [14] applied the Social Communication Questionnaire (SCQ) in a cohort of 21 children with TSC and searched for relationships between SCQ measures and cognitive development. The authors found ASD in 52 % of the patients, which correlated with intellectual functioning and AEDs number. We applied the same tool in our population. The use of the SCQ allowed us to identify patients with ASD symptomatology and to better characterize the severity of the psychiatric symptomatology. Furthermore, we correlated the SCQ scores with clinical features that could be implicated in disease modification.

The rate of ASD in TSC varies widely in published reports (from 17 % to 63 %). In our group of patients SCQ scores suggestive of ASD were found in 40.5 %, in line with recent literature data [8]. We also confirmed that in patients with TSC there is no gender difference for ASD [16]. A major limit of this study is that we couldn’t perform ADI/ADOS in all patients, currently considered the gold standard for the diagnosis of ASD [17]. However, all the diagnoses were confirmed by direct clinical observation, and we found no false positive and false negative results in our cohort, possibly due to the relatively small sample size. Therefore, although the SCQ should be considered a screening and not a diagnostic tool for ASD, it proved to be a reliable means for discriminating between patients with and without ASD in our sample.

With respect to ASD severity, the higher score on SCQ, the higher the likelihood that the child exhibits increased ASD symptomatology [18]. The scores were equally distributed in our sample, indicating that the degree of ASD in TSC patients may have a large variability. Also the clinical observation of these patients confirmed the absence of a uniform degree of severity. Interestingly, a narrow diagnosis of autism was identified in 35 % of patients. Furthermore, we found that patients with ASD were significantly different from patients without ASD with respect to the reciprocal social interaction domain and to the language/communication domain, while the restricted, repetitive, and stereotyped behaviors and interests domain did not reach significance between the two groups. In our experience we rarely noticed stereotypies and repetitive behaviors in these patients indeed, suggesting that in TSC patients with ASD this area might be less compromised than the others. However, further studies including a larger number of patients and using diagnostic tools for autism should be performed to better clarify this finding.

Regarding risk factors for ASD, epilepsy certainly plays a major role, since all the patients with ASD had epilepsy, and mean age of seizure onset was significantly lower in patients with ASD compared to patients without ASD (7.9 months vs 16.9 months). This finding further supports the association between epileptic activity and specific aspects of brain development, such as social intelligence and other cognitive skills involved in the pathophysiology of ASD [7].

Impaired neural connectivity rather than a localized deficit has been demonstrated to be responsible for the pathophysiological process of ASD [19]. This applies also to TSC patients, in which aberrant connectivity was demonstrated in normal-appearing perituberal cortex [20]. As a consequence of this new interpretation of the TSC brain, the role of tubers’ number and localization should be reconsidered. Interestingly, we couldn’t find any association between tubers localization and the SCQ score. In particular, patients with a pathologic SCQ score presented with frontal and temporal tubers as well as parietal and occipital, and temporal and parietal tubers were present in both patients with and without ASD.

Among the patients with a pathological SCQ score, those with seizure onset within the first year of life had the most severe signs of ASD, especially when epilepsy started with infantile spasms, thus confirming previous studies.

Early recognition of subtle focal seizures and infantile spasms, obtained throughout parental education and close EEG monitoring, allows prompt seizure control and can prevent epileptic encephalopathy and subsequent long-term cognitive and behavioral consequences, especially autism [21, 22]. Existing data are insufficient to support preventive therapy in infants with TSC without documented seizures, unless subclinical seizures are recorded [22]. The relationship between epileptic spasms and ASD has been recently supported by animal models, where *Tsc2* haploinsufficiency and developmental status epilepticus in *Tsc2*^+/−^ rats independently lead to social interaction deficits, suggesting the additive effect of seizures to increase the range of autistic-like behaviors in mutated animals [23].

A relationship between specific mutation types in patients with TSC and neurodevelopmental outcome is still debated. Numis et al. [8] found a higher prevalence of ASD in individuals carrying *TSC2* mutations, but they did not observe a significant association between ASD and mutation type in the *TSC2* gene. Also in our study the great majority of patients with ASD had a mutation in *TSC2* (88.2 %), 11.8 % had a mutation in *TSC1*, while no NMI patient had a pathological score. Furthermore, our results showed that TSC1 patients have less often a pathologic SCQ score (*p* = 0.05), while no difference was found in TSC2 patients. Recently, Huang et al. [24] found a relationship between ASD and nonsense mutations in the *TSC2* gene in a small group. Among our patients with truncating mutations, 12 had a pathologic SCQ score and 11 a normal score, so we couldn’t confirm that finding. In general, it was not possible to find a correlation between mutation subtypes and SCQ score, probably also due to the small sample size.

Cognitive level appeared to be strictly correlated with the average SCQ score, which tended to be progressively higher in patients with a worse ID. Furthermore, all the patients with a normal or borderline mental status didn’t have ASD. This data cannot be attributed to the tool itself because SCQ demonstrated a good specificity in diagnosing ASD independently of ID [13]. The linear relationship between ID and ASD emerging from our study has been recently demonstrated also by Van Eeghen et al. [9], but referring to Social Responsiveness. Moreover, it is very important to identify ASD in patients with ID because rehabilitation strategies can be modified and targeted according to the individual’s needs.

With respect to other neuro-psychiatric variables in TSC patients with ASD, we found a higher prevalence of sleep disorders, psychiatric symptoms and behavioral problems. Regarding sleep disorders in TSC, this issue has been poorly investigated [25, 26]. A sleep disorder has been reported in 30 % of patients with TSC, mainly insomnia, which showed a significant positive correlation with obstructive sleep apnea syndrome and restless legs scores [27].

Even though a higher prevalence of psychiatric symptoms in TSC patients rather than in general population is well known, few studies focused on this topic. The most frequently encountered formal diagnoses were anxiety disorders, mood disorders, adjustment disorders, ADHD and metal disorders not otherwise specified [2, 28]. Moreover, recent research focused on the possibility of recognizing a series of psychiatric symptoms, which can go along with the ASD core, such as self-injury, aggressiveness, stereotyped behaviors, and insistence on sameness [29–31]. With our study we confirm that psychiatric comorbidity is very common in patients with TSC, and should be managed with appropriate pharmacologic therapy in affected individuals.

Although our study is limited to 42 patients, it represents the largest cohort of TSC subjects assessed using the SCQ. Furthermore, we were able to precisely characterize the sample with respect to cognitive functioning and molecular genetics.

The patients’ selection due to the applicability of the SCQ could certainly represent a bias of the study, since a fairly high number of subjects with profound ID and adults not dependent on a caregiver were excluded. However, the selected patients underwent a solid clinical evaluation apart from the questionnaire.

## Conclusion

This study showed a prevalence of ASD of 40.5 %, confirming that patients with TSC have a high risk for this disorder. Early recognition of patients developing ASD symptomatology can bring them to early behavioral intervention focused on specific signs, and can address the need of global care requested by the families. The severity of ASD seems to have a notable variability in TSC patients. Risk factors for ASD in these patients are epilepsy, infantile spams, and mutations in *TSC2*, while the localization of tubers is not correlated to it.
